# Implementation of central line-associated bloodstream infection prevention bundles in a surgical intensive care unit using peer tutoring

**DOI:** 10.1186/s13756-017-0263-3

**Published:** 2017-10-02

**Authors:** Sang-Won Park, Suhui Ko, Hye-sun An, Ji Hwan Bang, Woo-Young Chung

**Affiliations:** 1 Infection Control Office, Boramae Medical Center, Seoul, Republic of Korea; 2 Department of Internal Medicine, Boramae Medical Center, Seoul National University College of Medicine, 20 Boramae-ro 5-Gil, Dongjak-gu, Seoul, 07061 Republic of Korea; 3 Intensive Care Units, Boramae Medical Center, Seoul, Republic of Korea

**Keywords:** Central line-associated bloodstream infection, Intensive care unit, Education, Intervention, Learning by teaching, Peer tutoring

## Abstract

**Background:**

Central line-associated bloodstream infections (CLABSIs) can be prevented through well-coordinated, multifaceted programs. However, implementation of CLABSI prevention programs requires individualized strategies for different institutional situations, and the best strategy in resource-limited settings is uncertain. Peer tutoring may be an efficient and effective method that is applicable in such settings.

**Methods:**

A prospective intervention was performed to reduce CLABSIs in a surgical intensive care unit (SICU) at a tertiary hospital. The core interventions consisted of implementation of insertion and maintenance bundles for CLABSI prevention. The overall interventions were guided and coordinated by active educational programs using peer tutoring. The CLABSI rates were compared for 9 months pre-intervention, 6 months during the intervention and 9 months post-intervention. The CLABSI rate was further observed for three years after the intervention.

**Results:**

The rate of CLABSIs per 1000 catheter-days decreased from 6.9 infections in the pre-intervention period to 2.4 and 1.8 in the intervention (6 m; *P* = 0.102) and post-intervention (9 m; *P* = 0.036) periods, respectively. A regression model showed a significantly decreasing trend in the infection rate from the pre-intervention period (*P* < 0.001), with incidence-rate ratios of 0.348 (95% confidence interval [CI], 0.98–1.23) in the intervention period and 0.257 (95% CI, 0.07–0.91) in the post-intervention period. However, after the 9-month post-intervention period, the yearly CLABSI rates reverted to 3.0–5.4 infections per 1000 catheter-days over 3 years.

**Conclusions:**

Implementation of CLABSI prevention bundles using peer tutoring in a resource-limited setting was useful and effectively reduced CLABSIs. However, maintaining the reduced CLABSI rate will require further strategies.

## Background

Central line-associated bloodstream infection (CLABSI) is one of serious healthcare-associated infections that cause increased medical costs, morbidity and mortality; however, CLABSIs have been prevented in many developed and developing countries using multifaceted approaches [1–5]. Several guidelines for the prevention of CLABSIs are available, but the core contents of the evidence-based recommendations are shared in common [6, 7]. Although the objectives of the CLABSI prevention guidelines are evident and simple, the implementation of these guidelines in clinical practices requires many factors to be well-coordinated. Heterogeneity in compliance or performance with the guidelines exists worldwide, and interventions have not always been successful [8]. The importance of infection control in healthcare settings for patient safety and quality of care cannot be emphasized enough, but the available resources including expert personnel, reimbursement systems and managerial support are not always sufficient to deal with many active issues in most healthcare facilities. Different strategies for different regional or institutional situations are needed for the successful implementation of CLABSI prevention guidelines.

The education of and feedback from healthcare workers are core components of implementing an intervention program. The education component should be organized in a manner that allows the healthcare workers to collaborate, learn from, and support each other. We used ‘learning by teaching method’-based education to implement CLABSI prevention bundles in a surgical intensive care unit (SICU) with a high CLABSI rate. This peer tutoring approach was intended to motivate the healthcare workers to actively participate in their own workplace problems and to develop a safety culture in the unit through the sharing of a common understanding.

## Methods

### Setting and subjects

This study was conducted in a surgical intensive care unit at a 767-bed tertiary hospital. The SICU had 15 beds, and most of the beds were occupied by patients from the neurosurgery and thoracic surgery departments. The patient to nurse ratio was 3:1. The SICU did not have a full-time intensivist responsible for overall clinical care but instead had a medical director whose main responsibility was administrative. All patients admitted to the SICU during the study period were included. The infection control office at the hospital comprised one infectious diseases physician who concurrently served as the director and one full-time and one part-time nurse.

### Study design and data collection

The primary goals of the intervention were to reduce CLABSIs in a SICU and to maintain the reduced rate. The secondary goals were to improve the perception of core knowledge related to CLABSI prevention and to retain compliance with the use of insertion and maintenance bundles. This study consisted of three periods, a pre-intervention period of 9 months (August 2011 to April 2012), an intervention period of 6 months (May 2012 to October 2012), and a post-intervention period of 9 months (November 2012 to July 2013). In addition, long-term follow-up of CLABSIs was performed for 3 years after 2013, and the infection control office intervened minimally during this period as specified in the ‘Intervention Program’ section below. The insertion bundle included hand hygiene, maximal barrier precautions, chlorhexidine skin antisepsis, and optimal catheter site selection with the subclavian vein identified as the preferred site for insertion that were explained in detail in previous reports [1, 9, 10]. The maintenance bundle included hand hygiene, catheter site dressing, hub care, and daily review of central line necessity [1, 9, 10]. For catheter site dressing, either sterile gauze or transparent semipermeable dressing was used and replaced every 2 days and 1 week respectively if not otherwise indicated. The site was disinfected with 2% chlorhexidine tincture. Before accessing catheter hubs, 70% alcohol was used for cleansing to reduce contamination.

Hand hygiene performance in the SICU was monitored weekly by an infection control nurse using the World Health Organization hand hygiene guide as a part of hospital-wide surveillance [11]. As the monitoring has a role of both measuring the performance and educating the healthcare workers on the spot, feedback about the hand hygiene performance was given to the health care workers immediately on the spot and monthly to each department. We only irregularly audit the hand hygiene performance by pre-trained unrelated external personnel to estimate the magnitude of Hawthorne effect for reference only. The perception of core knowledge was assessed for all nurses in the SICU. The survey consisted 20 questions total and was performed three times during the intervention period of 6 months. Residents or physicians in charge inserted catheter. The nurse in charge of each patient assisted the procedure and before the insertion, the nurse explained the checklist of insertion bundle and checked the adherence. The default of bundle was pointed out by the nurse in the middle of procedure and the persistence of default was recorded in the checklist. However, the nurse did not have the authority to stop the procedure.

CLABSIs were defined as laboratory-confirmed bloodstream infections in which a central line was in place for >2 calendar days on the date of event, and the line was in place on the date of the event or the day before [12]. To capture data for infections acquired during hospitalization only, the designation of CLABSI was considered valid only if the positive blood culture and clinical signs/symptoms of infection occurred at least 48 h after admission. The infection control office monitored the CLABSI events as well as compliance with use of the central line bundles. The number of pairs of blood cultures per 1000 patient-days was calculated during the study period to monitor the appropriate ordering practices of blood cultures [13].

### Intervention program

Regular team meetings between the infection control office and the SICU were held to implement and coordinate the CLABSI prevention bundles. The SICU team included the director of the unit, the head nurse and all of the working nurses. The infection control office designed the overall CLABSI prevention bundles and working programs and adjusted them based on the feedback received at the regular meetings.

**Fig. 1:**
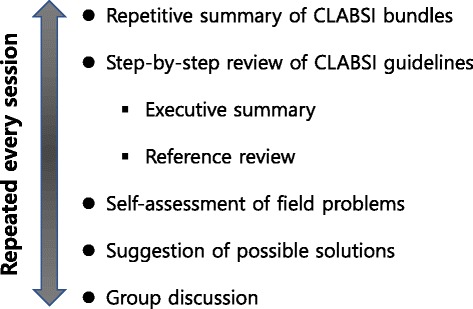
Contents of the peer tutoring educational session for nurses in the surgical intensive care unit

Implementation of the insertion and maintenance bundles was initiated through education, which provided working knowledge of the bundles, as well as individual and group feedback related to bundle use adherence. The educational sessions were conducted for all of the nurses in the SICU and were taught using the ‘learning by teaching’ or peer tutoring method in which the nurses themselves prepared and delivered the lecture contents (Fig. 1). After an initial introductory overview of the CLABSI bundles by the infection control office, these 30-min weekly educational sessions were held for 6 months (May 2012 – Oct 2012). The content of the lecture given by each nurse consisted of a repetitive summary of the core contents of the CLABSI bundles followed by a detailed review of one section of the published guidelines for the prevention of intravascular catheter-related infections, which was allocated to each lecturer [10]; a self-assessment of the current departmental problems in view of the reviewed content; and suggestions for possible solutions to these problems. The educational sessions were designed such that all of the SICU nurses served as lecturer, and the nurses had the opportunity to actively study and discuss the CLABSI-related problems. The framework of the lecture was suggested by the infection control office. The review sections included the guideline references to share their scientific basis. All of the participants were encouraged to discuss their problems or suggestions during every session. To educate doctors and encourage their cooperative compliance with the prevention bundles, separate monthly meetings were held between the infection control office and representative doctors from every clinical department that used the SICU because there was no intensivist or physician dedicated to patient care in the SICU. Indirect education for all of the doctors in clinical departments was performed through these representative doctors using presentation materials created by the infection control office. Regular group feedback and instant individual feedback were given pertaining to the performance of the CLABSI prevention checklists. The checklists used in the insertion and maintenance bundles were incorporated into the electronic medical record (EMR) system during the interventional period, and then automatic data collection in the infection control office and a short message alert service for the removal of central catheters were implemented. An all-in-one cart that had all of the necessary items for central line insertion was prepared.

After the intervention period, systematically fixed programs continued. There was weekly hand hygiene monitoring. The performance of checklists in the insertion and maintenance bundles of CLABSI prevention checked by the nurses in the SICU was monitored through EMR by the infection office, and the feedback for violation was provided to individual healthcare workers daily and clinical departments monthly. However, active educational meetings were not held any more. There was no systematic educational program about CLABSI bundle for new nurses of SICU, but they were expected to learn from their colleagues and fixed work pattern like daily checklists embedded in the EMR system. For new residents or physicians, educational material illustrated in a PowerPoint file was provided monthly through their e-mail. Active audit for the individual component of checklists on the spot was not performed.

### Statistical analysis

Comparisons of the CLABSI incidence rates during and after the intervention period with that of the baseline were analyzed by Poisson regression and are presented with 95% confidence intervals (CIs). All statistical tests were two-tailed, and *P* values <0.05 were considered significant (SPSS 22.0; SPSS Inc., Chicago, IL, USA).

## Results

**Fig. 2:**
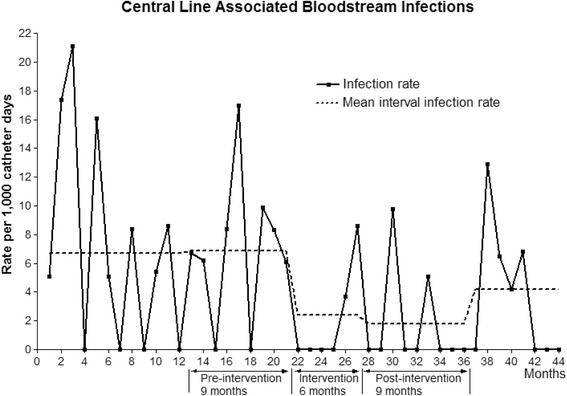
The trend of the central line-associated bloodstream infection (CLABSI) rate during the study periods. The broken lines indicate the mean CLABSI rate during each period

The rate of CLABSIs per 1000 catheter-days decreased from 6.9 infections in the pre-intervention period to 2.4 and 1.8 in the intervention (6 m; *P* = 0.102) and post-intervention (9 m; *P* = 0.036) periods, respectively (Table 1 and Fig. 2). The regression model showed a significantly decreasing trend in the infection rate from the pre-intervention period (*P* < 0.001) with incidence-rate ratios of 0.348 (95% confidence interval [CI], 0.98–1.23) in the intervention period and 0.257 (95% CI, 0.07–0.91) in the post-intervention period. However, after the 9 months post-intervention period, the yearly CLABSI rates reverted to 3.0–5.4 per 1000 catheter-days (Table 1).

**Table 1:** Central line utilization ratios and central line-associated blood stream infections in a surgical intensive care unit over 5 years

Period	Central line-days	Patient-days	Central line utilization ratio	^a^CLABSI events	^b^CLABSI rate
Pre-intervention, 9 m	1734	3273	0.53	12	6.9
Intervention, 6 m	1245	2147	0.58	3	2.4
After intervention,					
9 m	1684	3207	0.53	3	1.8
1st year (12 m)	1994	3920	0.51	9	4.5
2nd year (12 m)	2030	4137	0.49	6	3.0
3rd year (12 m)	2222	4657	0.48	12	5.4

^a^ *CLABSI* central line-associated bloodstream infection

^b^ *CLABSI rate* CLABSI events per 1000 central line-days

Adherence to each component in the insertion bundle reached 100% in the 5th month. Adherence to each component in the maintenance bundle reached 100% in the 2nd month. The absolute amount of central line use increased gradually but central line utilization ratio taking patient-days into account has been steadily decreasing since the initial rise during intervention period from 0.58 to 0.48 (Table 1). The places of central line insertion during intervention (6 m) and post-intervention (9 m) periods were operating room (58.6%), interventional radiology (20.8%), SICU (16.4%), emergency room (3.5%) and general ward (0.7%). The awareness of core knowledge about CLABSI prevention practices during the interventional period of 6 months increased, with scores of 15.8 (1st month), 17.1 (3rd month), and 18.9 (6th month) points out of a total of 20 points for the 20-question assessment. The performance of hand hygiene which was monitored weekly were 93.4% (range, 92–96), 89.7% (range, 79–97) and 90.9% (range, 83–96) during the pre-intervention, intervention and post-intervention periods, respectively.

Twenty pathogens were responsible for the CLABSIs during the 3 study periods, which totaled 24 months (Table 2). In the pre-interventional period, the predominant causative organisms were *Enterococcus* species (20%), *Acinetobacter baumannii* (20%), and coagulase-negative staphylococci (10%); one case each (5%) of *Staphylococcus aureus*, viridans streptococci, *Pseudomonas aeruginosa* and *Stenotrophomonas maltophilia* also occurred. In the intervention and post-intervention periods, *Candida* was the most common pathogen (4/6, 66.7%) of the 6 total causative agents, though the total number of infections was small. The *Candida* cases were not related to neutropenia. The mean pairs of blood cultures per 1000 patient-days during study periods were 201 (pre-intervention), 190 (intervention), and 196 (post-intervention).

**Table 2:** Microorganisms responsible for the central line-associated bloodstream infections during the three study periods

Organisms	Pre-intervention	During intervention	Post-intervention
	(9 m, *n* = 12)	(6 m, *n* = 3)	(9 m, n = 3)
Gram-positive bacteria			
*Staphylococcus aureusv*	1^a^	·	·
Coagulase-negative staphylococci	2^a^	·	1
viridans streptococci	1	·	·
*Enterococcus* species	4	·	·
*Corynebacterium* species		1	
Gram-negative bacteria			
*Acinetobactera baumannii*	4^a^	·	·
*Pseudomonas aeruginosa*	1	·	·
*Stenotrophomonas maltophilia*	1	·	·
Fungi			
*Candida albicans*	·	2	1
*Candida parapsilosis*	·	·	1

^a^Two cases were polymicrobial with *A. baumannii + S. aureus* and *A. baumannii* + coagulase-negative staphylococci, respectively

## Discussion

Our intervention to reduce CLABSIs by implementing CLABSI prevention bundles using peer tutoring was effective during the interventional period of 6 months and a post-interventional period of approximately 1 year. However, without continuous active interactions and dominating internal governance, the virtuous reduction of CLABSIs was not sustained. Our educational method had several advantages. Each healthcare worker had the opportunity to voice her/his own work-related problems, to hear from other colleagues, to understand the principles behind their routine activities and to receive responses to their problems. These factors minimized resistance to the introduction of a new job pattern. The purpose of the educational sessions was to motivate the healthcare workers, to provide them with expert knowledge for practical use and to improve knowledge retention over longer time periods. Determining the most effective educational method has been an area of utmost interest for a long time. Generally, active participatory education is more effective than passive learning. ‘Learning by teaching’ or peer tutoring is one of the active participatory educational methods in which the core idea is to have a pair or group of students teaching the majority of topics to their classmates in a way that encourages their classmates’ active participation and communication in the best possible way [14]. ‘Learning Pyramids’ have shown that learners can retain approximately 90% of the content of a subject matter when they teach the material to someone else.

The commitment of the infection control staffs to coordinate the overall program and the presence of governing leadership in the unit to maintain a level of practice quality were basic components of the intervention. Regardless of the strong positive aspects of our educational approach, the intervention failed to sustain a reduced CLABSI rate beyond 9 months post-intervention. Several complex factors seem to be responsible for this lack of sustainability. High job turnover in the unit coupled with the lack of a continuing education system for new nurses and doctors after the intervention period likely weakened the effectiveness of the prevention protocols. The turnover rates of nurses in the SICU were 24.9% and 59.5% due to resignation, leave of absence and rotation during intervention (6 m) and post-intervention (9 m) periods. Thereafter, the rate was 39.1% - 46.8% annually for 3 years. Interns or residents had a rotational program every 1 or 2 months among 3 affiliated family hospitals. The patient-to-nurse ratio of 1:3 was much higher than the previously reported rate of 1:2 which was a risk factor for CLABSIs [15]. Moreover, there was no dedicated intensivist in the SICU who could have led and maintained the program at the hospital.

Successful large-scale interventions with sustained reductions in CLABSIs have been reported worldwide [2, 3, 5, 16, 17]. In South Korea, there have been a few single center or small-scale trials to reduce CLABSIs [18–21]. The only recent multicenter interventional study involving 58 ICUs in 26 hospitals funded by the Korea Centers for Disease Control and Prevention resulted in a CLABSI rate of 2.23 infections per 1000 catheter-days, which did not significantly improve the pre-interventional rate of 2.09 infections per 1000 catheter-days [22]. There have been no reports about sustaining a reduced CLABSI rate in South Korea. The distribution of CLABSI pathogens in the pre-interventional period was similar to recent Korean National Healthcare-associated Infections Surveillance System (KONIS) data from 166 ICUs, which showed *A. baumannii* (14.6%) as second most common pathogen of CLABSI in 2013 [23]. The high number of *A. baumannii* infections in our study might reflect high *A. baumannii* infection/colonization in the SICU. Regarding relatively higher frequency of candida infection during the intervention and the immediate post-intervention periods, there were no changes of antibiotics prophylaxis or strategies of treatment of surgical site infection. The only systematic change was a use of 2% chlorhexidine tincture for skin disinfection before catheter insertion and catheter site dressing instead of 10% povidone-iodine as a CLABSI bundle component. Whether the chlorhexidine had weaker effect on candida that on other bacterial pathogens requires further data due to the small absolute number of candida infections in our study, but previous studies about CLABSI have not supported the role of chlorhexidine in candida infections.

There are diverse institutional situations and cultures that deal with infection control programs and patient safety issues. Although standard CLABSI prevention guidelines are well-known, their translation into clinical practice needs to be individualized according to regional or institutional feasibility. Our educational method was efficient in the initial implementation of the program and effectively reduced the CLABSI rate for a short time period of approximately one year. To maintain the reduced rate, a multifaceted integrative approach must be continued [15]. Intra-departmental or intra-unit effort hardly maintained the effects of the program over a longer time period. Our approach may not be effective in certain situations, but it deserves to be considered by institutions implementing a CLABSI prevention program for the first time, especially in resource-limited settings.

Our study has some limitations. First, as this was a single-center study, the effectiveness of our approach may not be generalizable. However, one institutional success may provide a good model for generalization, as has been shown previously [1, 24]. Second, we did not perform a comparative study to prove the effectiveness of the peer tutoring method. However, from a practical point of view, it was a useful tool to guide the program as it received the cooperation of the participants and improved the understanding between healthcare workers. Third, the indirect educational approach implemented for doctors in the SICU might have weakened the effect of the intervention. As residents from each clinical department who were mainly involved in the insertion procedure in the SICU rotated at 1 or 2 months interval among 3 family hospitals, integrative successive group education and follow-up assessment were impractical. And maintenance bundles were mainly related to the nurses. So, we decided to educate indirectly the doctors through preceptors in each department who had regular meetings with infection control office. The doctors had individual feedbacks on the spot in the SICU for their performance. Fourth, the performance of insertion bundle might be incomplete. Operating room was the most frequent place of central line insertion, but fully adopted the insertion bundle only after intervention period. They had been using a similar protocol except for skin disinfection with 10% povidone-iodine and a small sized drape. Emergency room (3.5%) and general ward (0.7%) had no insertion bundle though the number was small.

## Conclusions

A peer tutoring educational method was useful for the implementation of CLABSI prevention bundles in a SICU and effectively reduced infection rates for a short time period of approximately 1 year. This approach is applicable for hospitals with limited resources that are trying to initiate prevention bundles. However, to maintain the reduced CLABSI rates, resources support and multifaceted cooperative approaches may be essential.

## Abbreviations

CIs: Confidence intervals
CLABSI: Central line-associated bloodstream infection
EMR: Electronic medical record
SICU: Surgical intensive care unit

## References

[1] Pronovost P, Needham D, Berenholtz S, Sinopoli D, Chu H, Cosgrove S, Sexton B, Hyzy R, Welsh R, Roth G (2006). An intervention to decrease catheter-related bloodstream infections in the ICU. N Engl J Med.

[2] Bion J, Richardson A, Hibbert P, Beer J, Abrusci T, McCutcheon M, Cassidy J, Eddleston J, Gunning K, Bellingan G (2013). 'Matching Michigan': a 2-year stepped interventional programme to minimise central venous catheter-blood stream infections in intensive care units in England. BMJ Qual Saf.

[3] Berenholtz SM, Lubomski LH, Weeks K, Goeschel CA, Marsteller JA, Pham JC, Sawyer MD, Thompson DA, Winters BD, Cosgrove SE (2014). Eliminating central line-associated bloodstream infections: a national patient safety imperative. Infect Control Hosp Epidemiol.

[4] Yaseen M, Al-Hameed F, Osman K, Al-Janadi M, Al-Shamrani M, Al-Saedi A, Al-Thaqafi A. A project to reduce the rate of central line associated bloodstream infection in ICU patients to a target of zero. BMJ Qual Improv Rep. 2016. 10.1136/bmjquality.u212545.w4986. 10.1136/bmjquality.u212545.w4986 PMC4994091 27559470

[5] Marsteller JA, Sexton JB, Hsu YJ, Hsiao CJ, Holzmueller CG, Pronovost PJ, Thompson DA (2012). A multicenter, phased, cluster-randomized controlled trial to reduce central line-associated bloodstream infections in intensive care units. Crit Care Med.

[6] Latif A, Halim MS, Pronovost PJ (2015). Eliminating infections in the ICU: CLABSI. Curr Infect Dis Rep.

[7] Ling ML, Apisarnthanarak A, Jaggi N, Harrington G, Morikane K, Thu le TA, Ching P, Villanueva V, Zong Z, Jeong JS, Lee CM (2016). APSIC guide for prevention of central line associated bloodstream infections (CLABSI). Antimicrob Resist Infect Control.

[8] Valencia C, Hammami N, Agodi A, Lepape A, Herrejon EP, Blot S, Vincent JL, Lambert ML (2016). Poor adherence to guidelines for preventing central line-associated bloodstream infections (CLABSI): results of a worldwide survey. Antimicrob Resist Infect Control.

[9] Marschall J, Mermel LA, Classen D, Arias KM, Podgorny K, Anderson DJ, Burstin H, Calfee DP, Coffin SE, Dubberke ER (2008). Strategies to prevent central line-associated bloodstream infections in acute care hospitals. Infect Control Hosp Epidemiol.

[10] O'Grady NP, Alexander M, Burns LA, Dellinger EP, Garland J, Heard SO, Lipsett PA, Masur H, Mermel LA, Pearson ML (2011). Guidelines for the prevention of intravascular catheter-related infections. Clin Infect Dis.

[11] World Health Organization. WHO Guidelines on hand hygiene in health care. http://apps.who.int/iris/bitstream/10665/44102/1/9789241597906_eng.pdf. 2009. Accessed 1 Aug 2017.

[12] Centers for Disease Control and Prevention. Bloodstream infection event (central line-associated bloodstream infection and non-central line-associated bloodstream infection). http://www.cdc.gov/nhsn/pdfs/pscmanual/4psc_clabscurrent.pdf. 2017. Accessed 1 Aug 2017.

[13] Baron EJ, Weinstein MP, Dunne WM, Yagupsky P, Welch DF, Wilson DM (2005). Cumitech 1C: Blood cultures IV.

[14] Grzega J, Schöner M (2008). The didactic model LdL (Lernen durch Lehren) as a way of preparing students for communication in a knowledge society. J Educ Teach.

[15] Fridkin SK, Pear SM, Williamson TH, Galgiani JN, Jarvis WR (1996). The role of understaffing in central venous catheter-associated bloodstream infections. Infect Control Hosp Epidemiol.

[16] Palomar M, Alvarez-Lerma F, Riera A, Diaz MT, Torres F, Agra Y, Larizgoitia I, Goeschel CA, Pronovost PJ, Bacteremia Zero Working G (2013). Impact of a national multimodal intervention to prevent catheter-related bloodstream infection in the ICU: the Spanish experience. Crit Care Med.

[17] Pronovost PJ, Watson SR, Goeschel CA, Hyzy RC, Berenholtz SM (2016). Sustaining reductions in central line-associated bloodstream infections in Michigan intensive care units: a 10-year analysis. Am J Med Qual.

[18] Kim OS, Kim SM (1999). Prevention of central venous catheter-related infections. Korean J Nosocomial Infect Control.

[19] Yoo S, Ha M, Choi D, Pai H (2001). Effectiveness of surveillance of central catheter-related bloodstream infection in an ICU in Korea. Infect Control Hosp Epidemiol.

[20] Lee DH, Jung KY, Choi YH (2008). Use of maximal sterile barrier precautions and/or antimicrobial-coated catheters to reduce the risk of central venous catheter-related bloodstream infection. Infect Control Hosp Epidemiol.

[21] Yoo S, Jung SI, Kim GS, Lim DS, Sohn JW, Kim JY, Kim JE, Jang YS, Jung S, Pai H (2010). Interventions to prevent catheter-associated blood-stream infections: A multicenter study in Korea. Infect Chemother.

[22] Yoon YK, Lee SE, Seo BS, Kim HJ, Kim JH, Yang KS, Kim MJ, Sohn JW (2016). Current status of personnel and infrastructure resources for infection prevention and control programs in the Republic of Korea: A national survey. Am J Infect Control.

[23] Choi JY, Kwak YG, Yoo H, Lee SO, Kim HB, Han SH, Choi HJ, Kim HY, Kim SR, Kim TH (2016). Trends in the distribution and antimicrobial susceptibility of causative pathogens of device-associated infection in Korean intensive care units from 2006 to 2013: results from the Korean Nosocomial Infections Surveillance System (KONIS). J Hosp Infect.

[24] Berenholtz SM, Pronovost PJ, Lipsett PA, Hobson D, Earsing K, Farley JE, Milanovich S, Garrett-Mayer E, Winters BD, Rubin HR (2004). Eliminating catheter-related bloodstream infections in the intensive care unit. Crit Care Med.

